# The Detection of Stone within the Urinary Organs

**Published:** 1907-08-17

**Authors:** Reginald Harrison

**Affiliations:** Past Vice-President and Hunterian Professor, Royal College of Surgeons


### THE DETECTION OF STONE WITHIN THE URINARY ORGANS.

### Some Clinical Remarks on Modern Methods.

By REGINALD HARRISON, F.R.C.S., Past Vice-President and Hunterian Professor, Royal College of Surgeons.

Amongst the various circumstances which have contributed to the great improvement in the treatment of stone as affecting the urinary organs must be included the means now at our disposal for detecting its presence when the possibility of its spontaneous escape no longer exists.

The risk attending the removal of a stone, for instance, from the bladder may be fairly estimated by the size the calculus has by lapse of time been allowed to attain. The mortality connected with such operations may therefore be stated as varying from nil to that which can only be expressed as somewhere approaching double figures. Hence the importance of the early detection of stone wherever it may be situated.

A stone imprisoned within the kidney is likely to lead in the course of time to the destruction of this organ, and the same thing happens when a calculus by blocking the ureter leads to the production above of a pyo- or hydro-nephrosis; so, too, a bladder which fails to expel a stone that has dropped from the kidney and may be detained by a large prostate, or otherwise, contains a source of cystitis so long as the stone is undiscovered and is allowed to remain.

It is not a very long time since the only method for detecting a stone within the bladder was by means of a metal instrument known as a sound. This is useless if the bladder is so distorted by an enlarged prostate, or by sacs or pouches, for without unwarrantable force it is then impossible to bring the beak of the sound in contact with the stone.

In the case of children and adults and patients where stone is uncomplicated with any abnormal physical conditions of the parts, a number 8 or 9 metal sound which will move about easily is still a ready and safe method of diagnosis. A well-shaped sound should under capable guidance touch the entire surface of a normal bladder, though many instruments sold for this purpose do not fulfil that condition. There are two spots immediately outside the boundary-line of a normal bladder where stones are undetectable by the sound. I refer to the orifices of the ureters. In abnormally shaped bladders where, for instance, the prostate may be greatly enlarged or its lining membrane sacculated and uneven, there are several spots where a stone cannot be felt with a sound even when an anæsthetic is used; and it is just in these very places that stones often locate themselves. Indeed, these more restful spots may be said to be favourable to the formation of stone, and are also out of the way of discovery. The electric cystoscope and the *x*-rays have contributed importantly to remove the difficulties connected with the diagnosis of stone in these positions.

Where a stone lies in a bladder sac or pouch it is almost impossible to feel it with the metal sound, and serious damage has not infrequently followed attempts to do so. Failing this and the *x*-rays, when there are still reasons for believing that a stone in the bladder may be so situated, there is no alternative but to open the bladder, when stone and sac may both be dealt with directly. It is not to be inferred that the employment of the *x*-rays in these as well as in other conditions where urinary stone is suspected is an infallible test. This is not the case. A trial of the rays should be made in all doubtful cases before an operation is proceeded with. Instances are not uncommon where unmistakable evidence of stone has thus been unexpectedly afforded. On the other hand, the failure of this test in diagnosing a stone does not negative an exploratory operation should other symptoms appear to justify it. A recent writer * on this subject has well said, "I am not at all inclined to underestimate the value of the newer methods of diagnosis, but I do not think it proper to subject a patient to cystoscopy and an *x*-ray examination, with perhaps ureteral catheterisation, as soon as one finds something wrong with the urine, without attempting to arrive at a diagnosis in the old orthodox way."

Instances have come under observation where the cystoscope has distinctly demonstrated the presence of small torpedo-shaped stones composed of uric acid or oxalate of lime tipped with white phosphates, lying just within the orifice of a ureter. In an observed instance a patient had suffered after exercise from symptoms of bladder stone for many months. He had been frequently sounded with negative results. On cystoscoping him under cocaine injection an appearance was found as stated; a white glistening point occupying the dimple of a ureter distinctly marked the presence of a stone. Shortly after this examination was made—in fact in a few hours—the patient expelled the calculus spontaneously, and all his symptoms, which had produced great discomfort and doubt, at once ceased and never recurred. Doubtless the manipulations connected with the use of the cystoscope or the preliminary distension of the bladder with water aided the stone to make its escape from a position it had occupied for some months. In many other similar cases the cystoscope has shown what was more or less covered by a prostatic flap or projection, and which a metal sound was unlikely to touch, and has enabled a diagnosis to be made. Thus radiography and electricity have considerably added to our resources in the diagnosis of bladder stones. They have also proved of great value in the detection of stone in the kidney and at points along the ureter. Further developments may be expected, and we may live to see the day when radiography in some form may render obsolete the somewhat disagreeable process (without an anæsthetic) of sounding for calculus with a metal instrument.

* Dr. Robertson, *Interstate Medical Journal, U.S.A.*, May 1907.

